# Supplementary figures and images for: Yeast heterochromatin regulators Sir2 and Sir3 act directly at euchromatic DNA replication origins
Source: PLoS Genet. 2018 May 24;14(5):e1007418. doi: 10.1371/journal.pgen.1007418 (PMC5991416; doi:10.1371/journal.pgen.1007418)

*CDC6*  
*SIR2*

*CDC6*  
*sir2Δ*

*cdc6-4*  
*SIR2*

*cdc6-4*  
*sir2Δ*

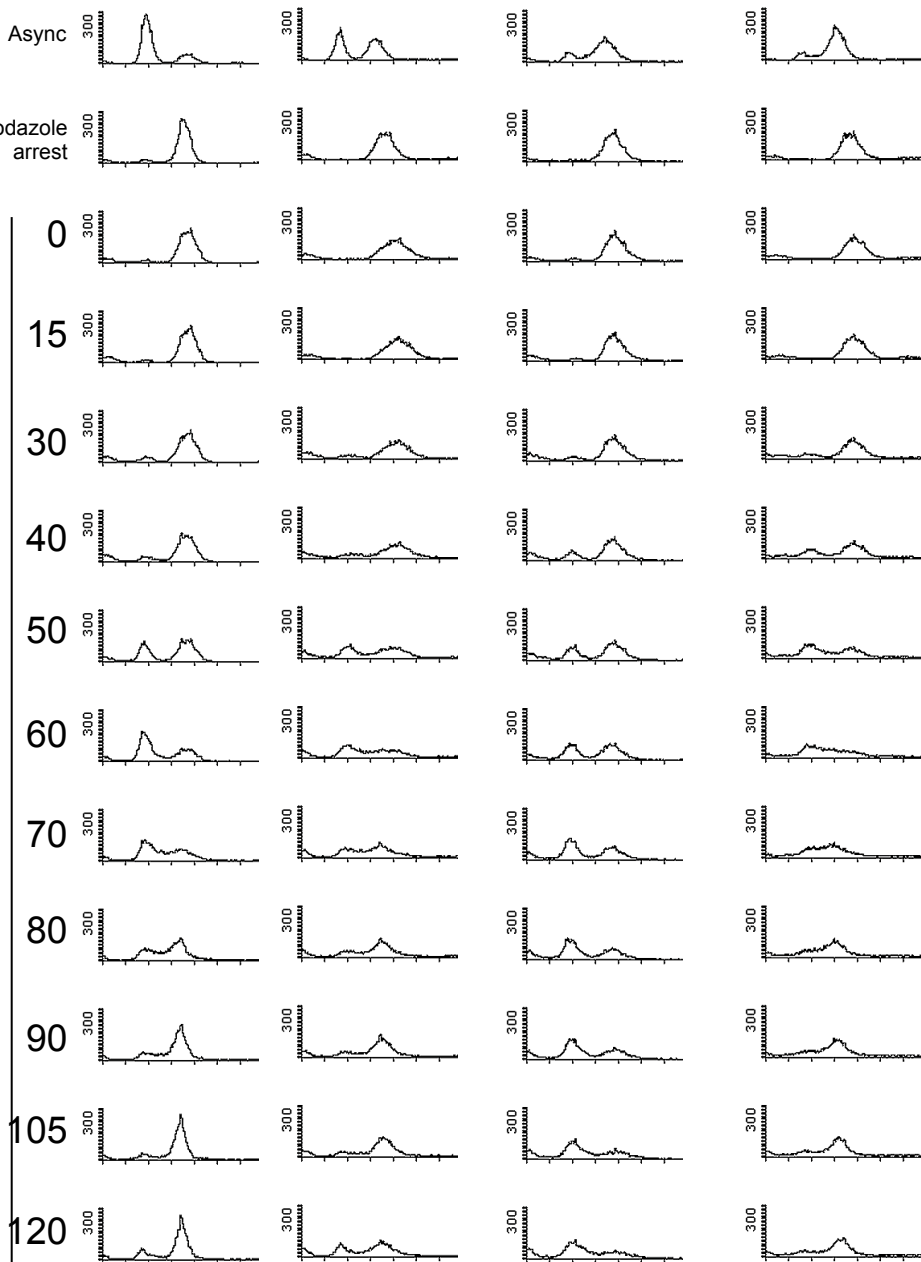

Supplement: S1 Fig — Formaldehyde was added to the CDC6 SIR2, CDC6 sir2Δ, cdc6-4 sir2Δ cell cultures at 55 minutes aftert nocodazole release. For cdc6-4 SIR2 cells, formaldehyde was added 105 minutes after nocodazole release. (PDF) [file pgen.1007418.s001.pdf]

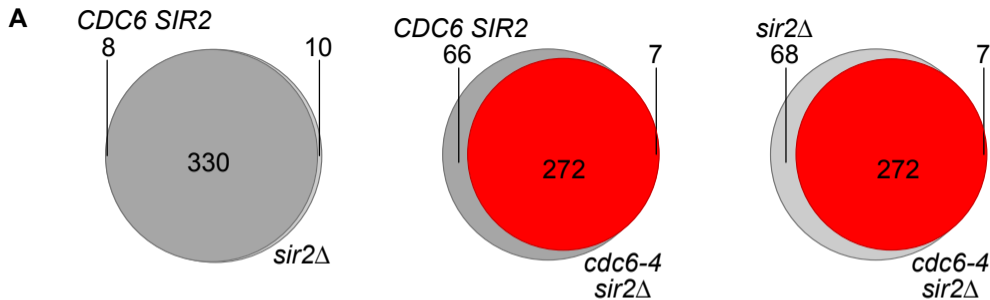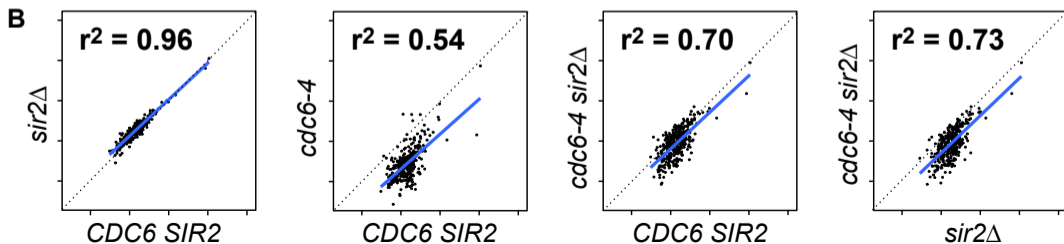

Supplement: S2 Fig — A. The MCM ChIP-Seq signals that identified confirmed origins in CDC6 SIR2 and CDC6 sir2Δ cells were compared. 340 confirmed origins were identified among the top 400 MCM peaks in CDC6 sir2Δ, while 338 were identified in CDC6 SIR2 cells. Among these, 330 were contained in both the CDC6 SIR2 and CDC6 sir2Δ data sets, indicating >97% overlap. For the top 400 peaks identified in the cdc6-4 sir2Δ, 279 identified confirmed origins. 272 of these were also found in either the CDC6 SIR2 or the CDC6 sir2Δ data sets, indicating that ~83% of confirmed origins otherwise defective in MCM association in cdc6-4 cells were rescued by the sir2Δ mutation. B. The areas defining the MCM ChIP-signals in CDC6 SIR2 cells were determined, and then the corresponding coordinates were used to determine the areas under the corresponding regions in the mutant cells. Comparison of CDC6 sir2Δ and CDC6 SIR2 data generated a strong correspondence, indicating that in otherwise wild type cells, a sir2Δ had a minimal effect on MCM distribution across origins. In contrast, comparison of the SIR2 cdc6-4 and CDC6 SIR2 data sets indicated a poor relationship between the areas of signals for origins, consistent with cdc6-4 abolishing virtually all MCM binding. However, the cdc6-4 sir2Δ cells generated areas more similar to that of wild type CDC6 SIR2 or CDC6 sir2Δ cells, consistent with the substantial amount of MCM binding rescue described in Fig 1 and in (A) of this figure. (PDF) [file pgen.1007418.s002.pdf]

**A**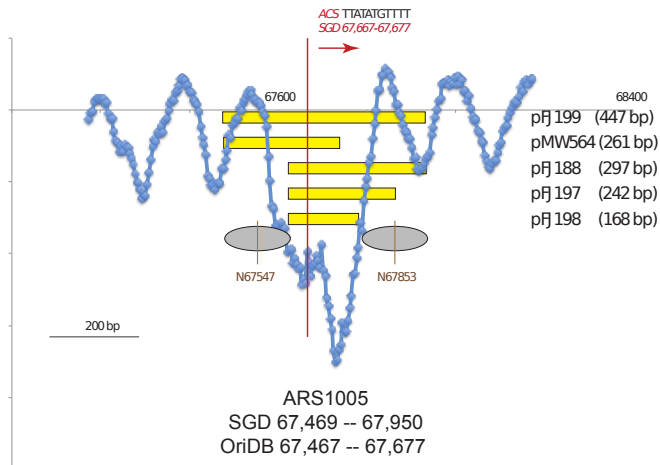**B**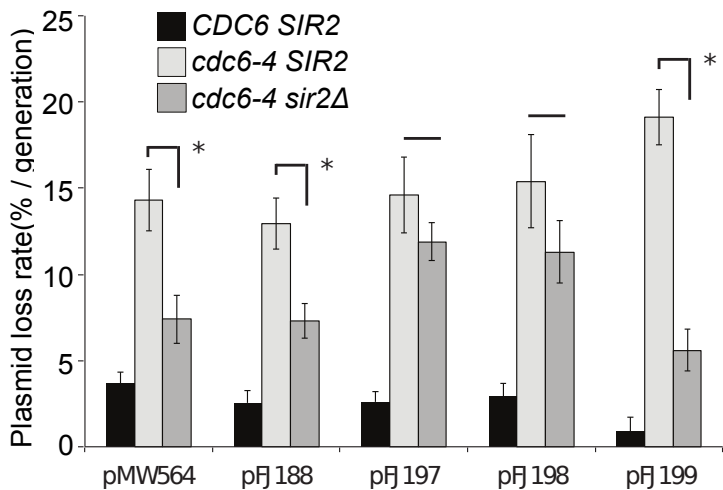

Supplement: S3 Fig — A. ARS1005 was an origin that showed substantial rescue of MCM binding (by ChIP-Seq) in cdc6-4 sir2Δ cells. The blue line indicates the trace of nucleosome occupancy at ARS1005 and the yellow boxes indicate the extent of ARS1005 sequences inserted in the CEN4 URA3 plasmid pARS1-WT, that replaced ARS1 as described (Chang et al. 2011, NAR). The ACS (ARS Consensus Sequence), the conserved 11-bp core sequence element within the binding site for yeast ORC, is indicated with a red line and was confirmed by mutation in pFJ199 and pMW564 contexts. B. The indicated plasmid clones were transformed into WT (M138), cdc6-4 (M386) and cdc6-4 sir2Δ strains (M922) and then assayed for plasmid stability over approximately 10 generations as described [23] (Crampton et al., 2008, Mol Cell). The high cdc6-4 plasmid loss rate of pFJ197 and pFJ198 (that did not contain chromosomal sequences overlapping positioned nucleosomes) were not significantly rescued by sir2Δ. In contrast, those plasmids that contained larger inserts of DNA that would contain one (pFJ188 and pMW564) or both (pFJ199) positioned nucleosomes were significantly rescued. (PDF) [file pgen.1007418.s003.pdf]

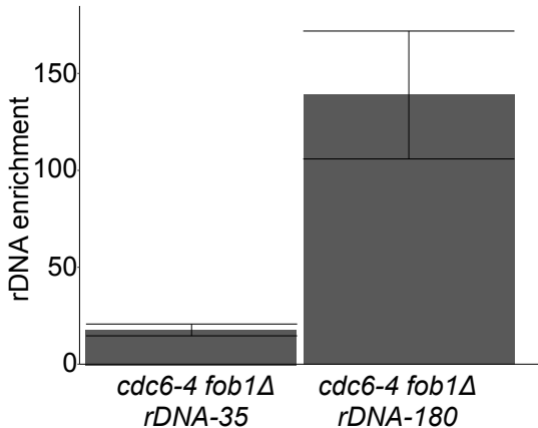

Supplement: S4 Fig — The cdc6-4 fob1Δ strains for the experiment in Fig 2D were generated by crossing a cdc6-4 FOB1 and CDC6 fob1Δ: HIS3 rDNA-35 (or fob1Δ fob1Δ: HIS3 rDNA-180, as appropriate, where rDNA-## indicates number of copies of the rDNA locus) parents. The resulting HIS3+ and temperature-sensitive haploids examined in Fig 2D were confirmed to be cdc6-4 by PCR of a 700 bp region followed by a digest with HhaI to generate ~600 and ~100 bp fragments. The rDNA copy number of these haploids was then confirmed by qPCR using the same primers described in Fig 2. The data show the enrichment values for the rDNA copy number of these strains, as indicated, for three technical replicates each and the error-bars indicate standard deviation for the three independent reactions. (PDF) [file pgen.1007418.s004.pdf]

H2AK5ac

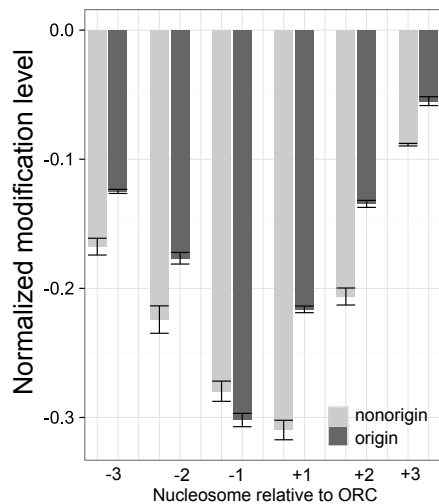

H3K14ac

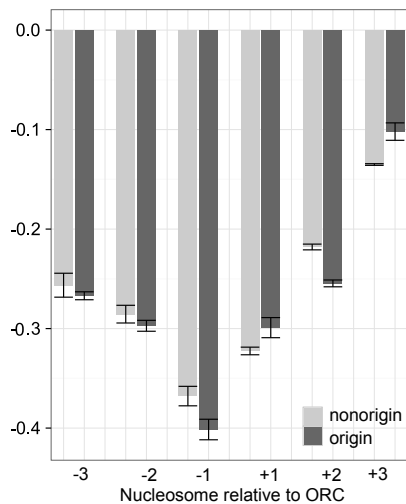

H3K18ac

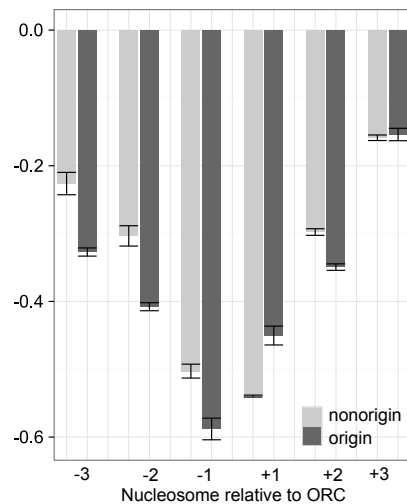

H3K23ac

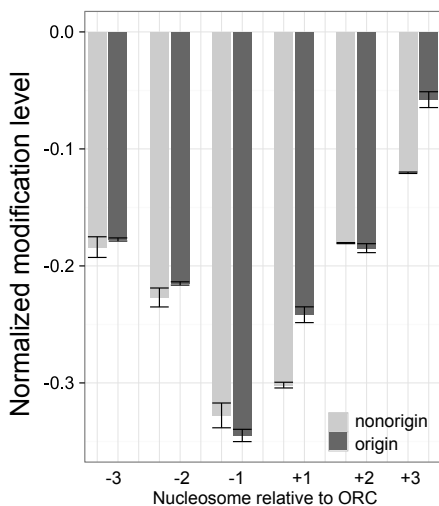

H3K27ac

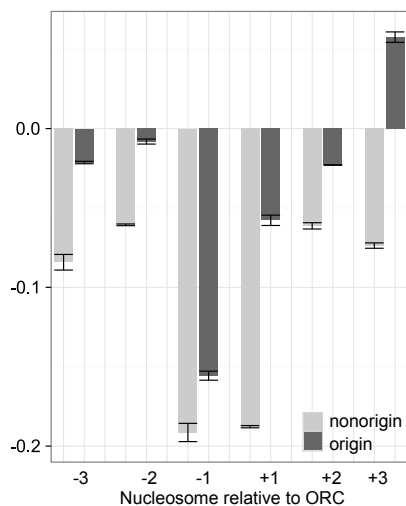

H3K4ac

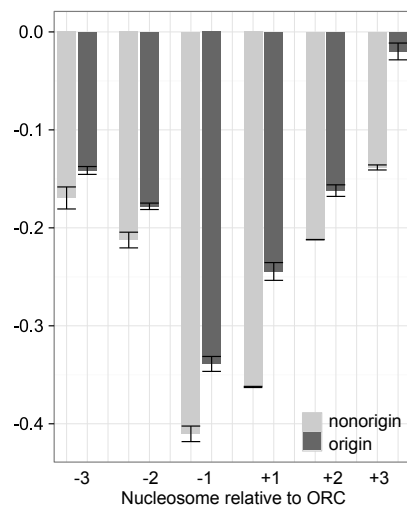

H3K56ac

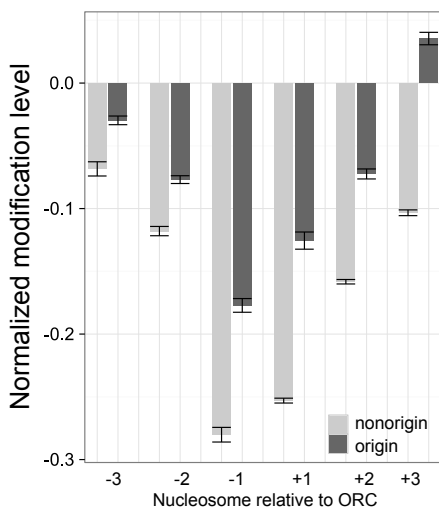

H4K12ac

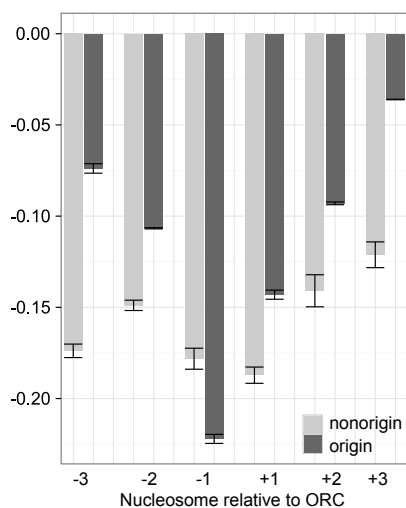

H4K5ac

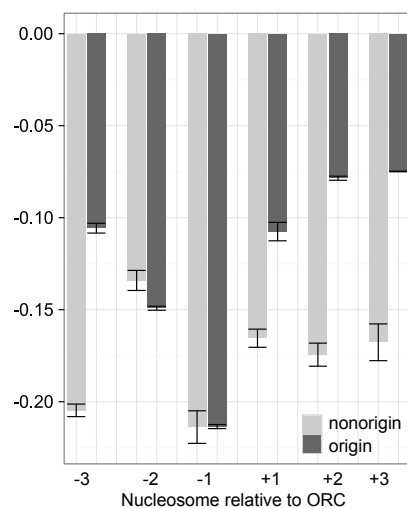

Supplement: S5 Fig — Most acetylation marks were depleted similarly from nucleosomes adjacent to origin and non-origin loci relative to the control intergenic loci. Normalized acetylation status was determined for each of the indicated acetylation marks on each of the indicated nucleosomes using the genome-wide histone modification atlas from [32] (Weiner et al., 2015) as described for Fig 3. (PDF) [file pgen.1007418.s005.pdf]

H4K16ac modification level

Nucleosome relative to ORC site

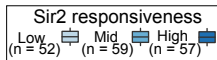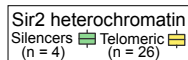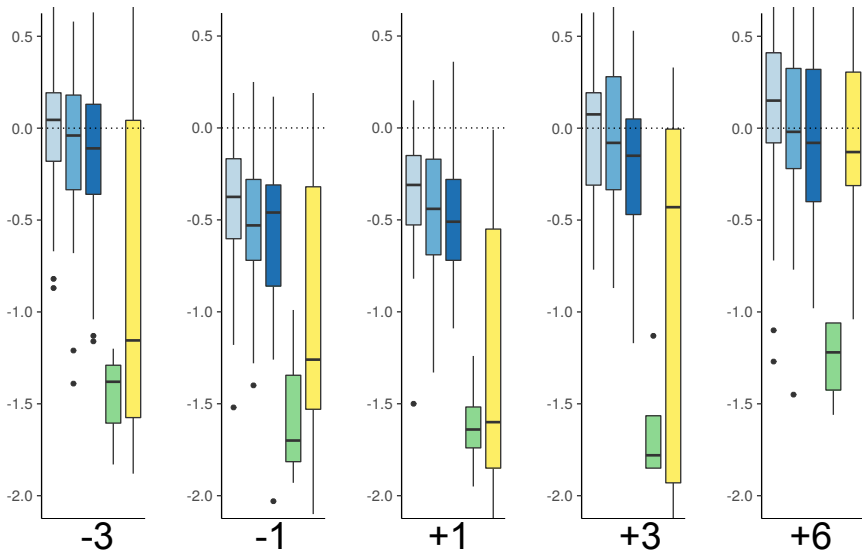

Supplement: S6 Fig — Log2 values for H4K16ac/total (input) ratios (y-axis) were determined for each indicated nucleosome (x-axis) for each origin group indicated (colored columns). The euchromatic origins were subdivided into three subgroups based on their SIR2-reponsiveness (i.e. the ratio of the MCM ChIP signal over the origin coordinates in cdc6-4 sir2Δ mutant cells to the MCM ChIP signal in wild type (CDC6 sir2Δ) cells.) The origin from within the HM SIR-heterochromatic loci (silencers) and from within the SIR-heterochromatic telomeres were also assessed for H4K16ac in this experiment. The “0” binding baseline was generated using all S. cerevisiae nucleotides (i.e. without pre-excluding nucleotides from within SIR-heterochromatin) using the method described in the main text. (PDF) [file pgen.1007418.s006.pdf]

**A**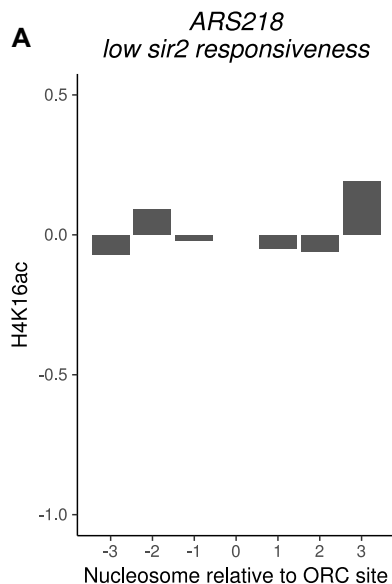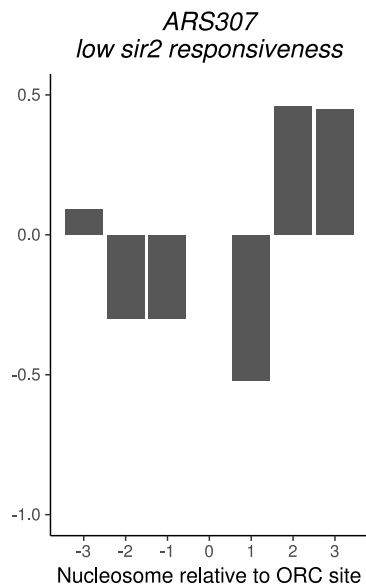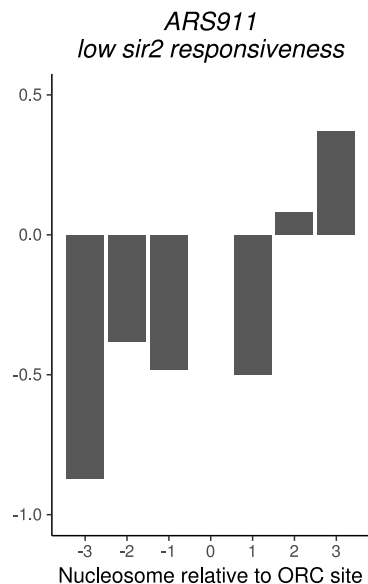**B**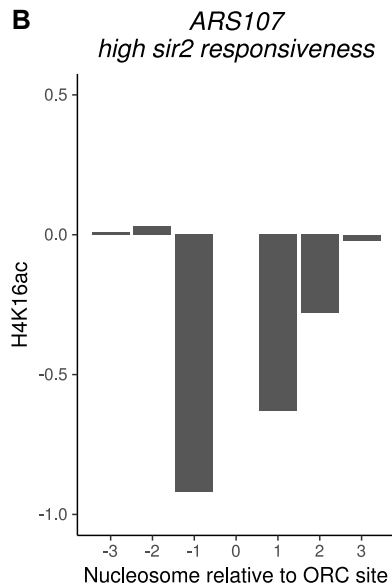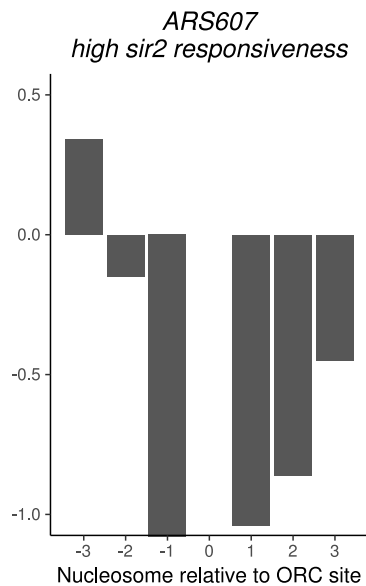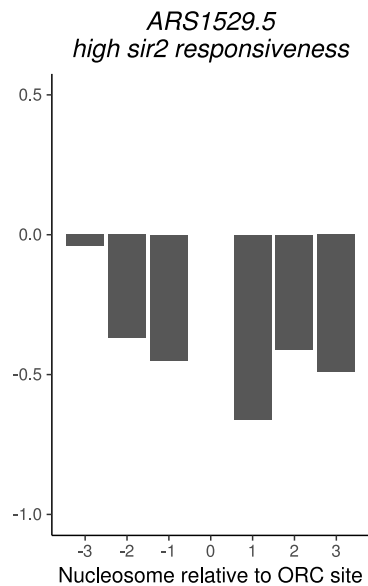

Supplement: S7 Fig — The H4K16ac status for the indicated origin-adjacent was determined as described for Fig 3D. A. Three origins selected from the quintile containing low SIR2-responsive origins as described for Fig 3E. B. Three origins selected from the quintile containing the high SIR2-responsive origins as described for Fig 3E. (PDF) [file pgen.1007418.s007.pdf]

**A**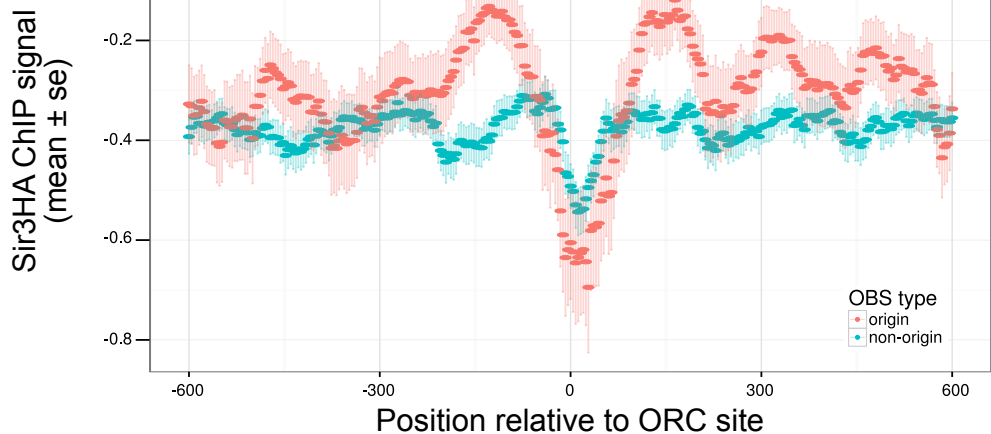**B**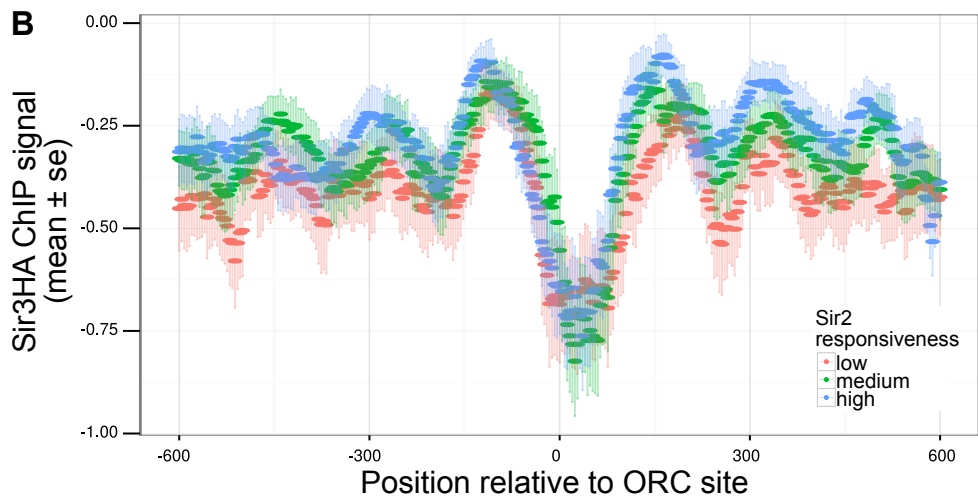**C**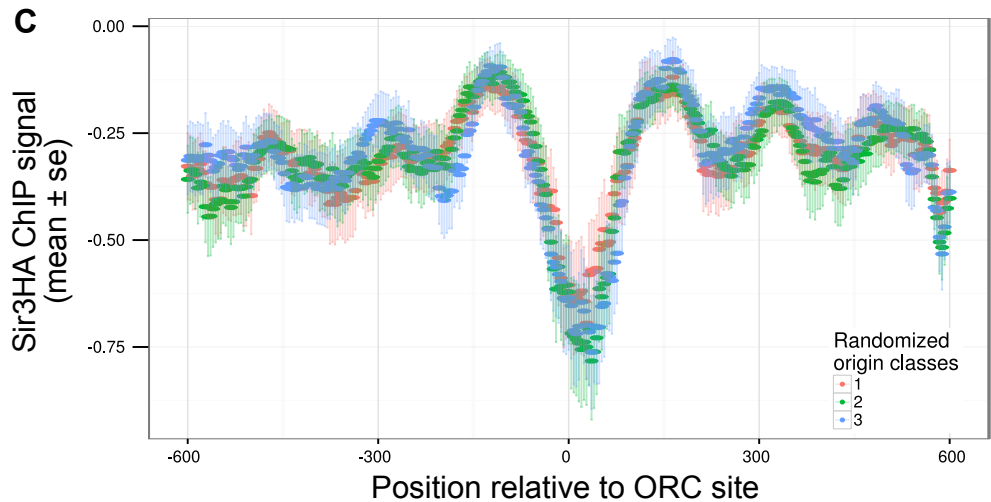

Supplement: S8 Fig — A. Sir3-3xHA ChIP-Seq signals at euchromatic origins and non-origin ORC-site loci relative to a baseline generated using all using all S. cerevisiae nucleotides (i.e. without pre-excluding nucleotides from within SIR-heterochromatin) using the method of [49] and as described in the main text. The data are from [50]. These data are for comparison to the Sir3 data shown in Fig 5C of the main text and indicated that excluding SIR-heterochromatin regions shifts the baseline for “0” Sir3 binding, as expected if the vast majority of stable Sir3 binding is confined to the telomeric and HM regions of the genome. However, the relative Sir3-3xHA ChIP-Seq signal differences between origins and non-origin ORC-site control loci are still apparent. B. Sir3-3xHA ChIP-Seq signals at the three groups of euchromatic origins that differ based on their SIR2-responsiveness as shown in Figs 3E and 5D. C. The SIR2-responsive origins comprising the three groups assessed in ‘B’ were randomized into three different sets and the Sir3-3xHA ChIP-Seq signals determined and plotted as in ‘B’. (PDF) [file pgen.1007418.s008.pdf]

WT H3-Q76A H3-Q76R H3-K79A H3-K79E H3-K79Q H3-K79R H3-T80A H3-D81A H3-D81K

25°C

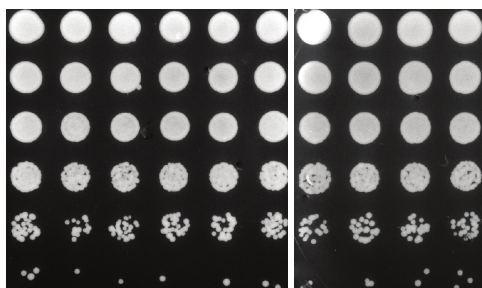

30°C

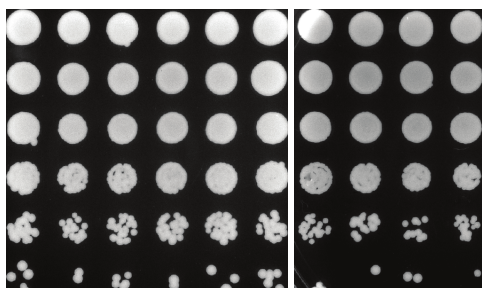

35°C

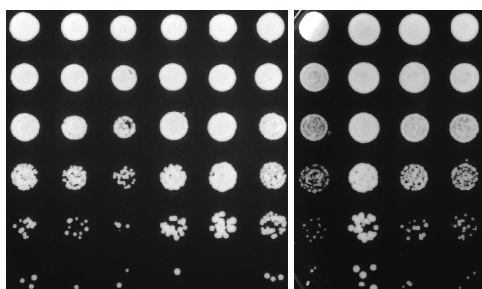

37°C

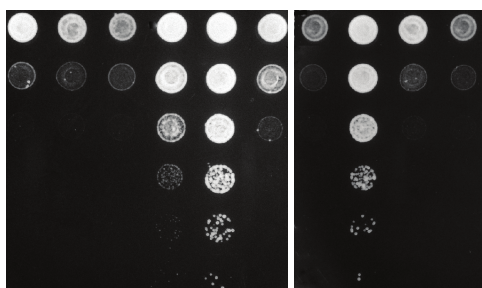

Residues near H3-K79

Supplement: S9 Fig — “WT” refers to cdc6-4. That is, all strains assessed are of the cdc6-4 genotype, as in Fig 6B. (PDF) [file pgen.1007418.s009.pdf]
